# An Analysis of Security System for Intrusion in Smartphone Environment

**DOI:** 10.1155/2014/983901

**Published:** 2014-08-05

**Authors:** Maya Louk, Hyotaek Lim, HoonJae Lee

**Affiliations:** ^1^Department of Ubiquitous IT, Graduate School of Dongseo University, Sasang-Gu, Busan 617-716, Republic of Korea; ^2^Division of Computer and Engineering Dongseo University, Sasang-Gu, Busan 617-716, Republic of Korea

## Abstract

There are many malware applications in Smartphone. Smartphone's users may become unaware if their data has been recorded and stolen by intruders via malware. Smartphone—whether for business or personal use—may not be protected from malwares. Thus, monitoring, detecting, tracking, and notification (MDTN) have become the main purpose of the writing of this paper. MDTN is meant to enable Smartphone to prevent and reduce the number of cybercrimes. The methods are shown to be effective in protecting Smartphone and isolating malware and sending warning in the form of notification to the user about the danger in progress. In particular, (a) MDTN process is possible and will be enabled for Smartphone environment. (b) The methods are shown to be an advanced security for private sensitive data of the Smartphone user.

## 1. Introduction

Malware applications inhabit the application store and market. It does not only intrude via downloading or installing activity, but also intrude via access to particular website and SMS. Juniper research finds that 80% of Smartphone device will remain vulnerable for cyberattacks through 2013 [1]. This happens although there is an increasing in customer awareness toward the issue of mobile security products. According to Juniper, there are several factors upon the cause of low level of adoption for security products. It is expected that, by 2018, 1.3 billion mobile devices including smart phones, feature phones, and tablets are fortified by mobile security devices, up from around 325 million this year. According to the study by the Department of Homeland Security and the Federal Bureau of Investigation, as the dominant mobile operating system, android is the primary target for malware attacks because there are many users who are still using the older versions of the software [1]. According to the government agencies, 79 percent of the existing malwares are threatening android mobile system while the rest are haunting the other mobile systems [2].

The growth rate for threats targeting mobile platforms has increased dramatically: 40,059 of the 46,415 modifications and 138 of the 469 mobile malware families were added to our database in 2012 [3].

99% of mobile malware detections in 2012 were targeting android devices. For the next two years, it is clear that android will become the dominant target for malware attacks. Android operating system has become the most common operating system and the most interesting system to be attacked by malware-maker. The formula stands as follows: “the most prevalent OS” + “installation of software from any source” = “the greatest number of threats” [3].

**Figure 1:**
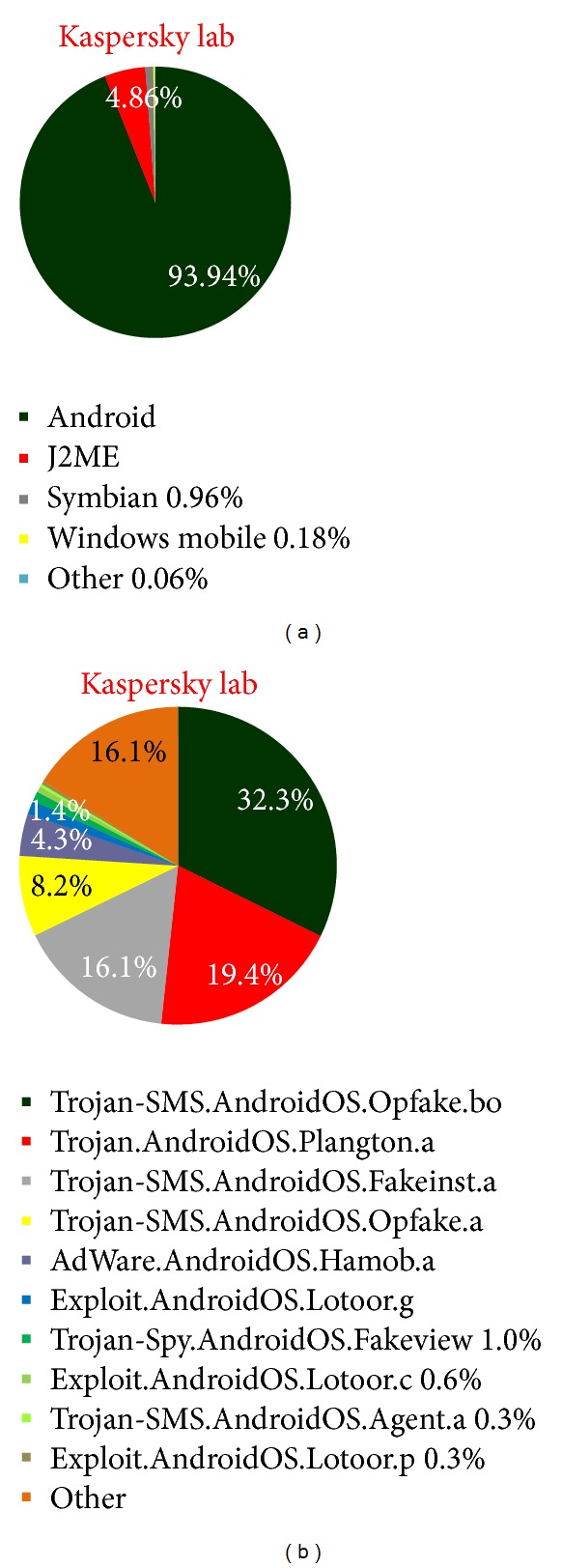
(a) Distribution of mobile threats by platform, 2004–2012; (b) the most frequently detected malicious programs targeting android.

**Figure 2:**
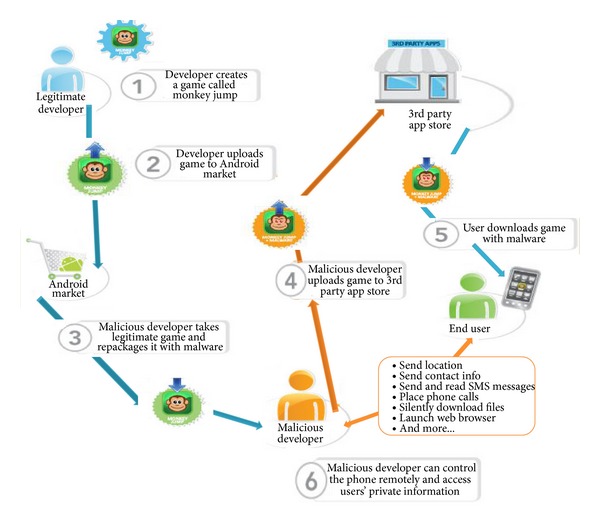
Repackaging applications process.

**Figure 3:**
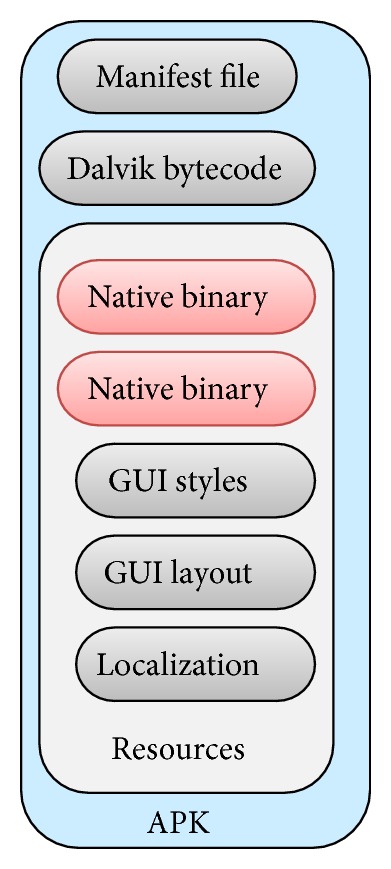
The android installation file containing malicious components.

**Figure 4:**
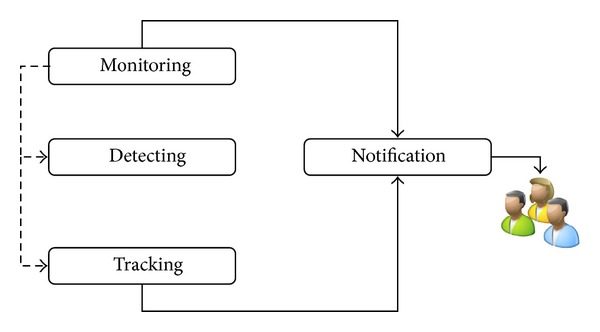
Outline of MDTN.

Based on the research by Kaspersky lab and Juniper research, Figure 1 shows the Most targeted Mobile Operating System by intruders is Android (Figure 1(a)) and the most malware injected by intruders through Android is Trojan-SMS.AndroidOS.Opfake.bo (Figure 1(b)), this confirmed from the result in Table 1 and Table 2, where android hold the largest market share (Table 1) and the biggest threats modification by intruders. Based on this, we propose a new approach to analyze the behavior of malware in Smartphone. The idea will be running in android environment. The idea consists of methods in monitoring, detecting malicious program inside the Smartphone, and tracking and notifying the user about the result and progress.  Figure 2 illustrates how intruder works for repackaging a malware application process and also in Figure 3 illustrates the android installation file containing malware components to the mobile's user. Based on this, MDTN is an interconnected process with two focuses. Malware application will be detected and any suspicious activity will be monitored in real time and notification will be sent to the user, all with the help of cloud computing system which is connected to the Smartphone for signature database.The outline of this work shows in Figure 4. The main contribution of this idea is methods in the form of MDTN which could be used by other researchers to track cyber intruders.

## 2. Related Work

There are a lot of researches about malware application up until 2013. Malware applications are being labeled (Kaspersky lab, Juniper research). Research about methods or species are also developed by several institutions (Cloud Security Alliance—CSA). According to CSA, malware could be deployed not only via website link, fake application, or smishing (SMS phishing), but also via Wi-Fi connectivity [4].

There are a lot of researchers who have been contributing ideas to improve security system to prevent data loss in mobile computing like Oliveira et al. via Honeypotlabsac, a virtual honeypot for android which emulate intrusion detection on services like telnet, http, and SMS [5].

Some researchers provide their own security model [6–8]. The permission-based security model is one of the most important security models in android devices. The user could grant or deny the installation and the application itself specifies which resources of the device need to be used. Analysis and enforcement of this permission-based model have been proposed by various researchers [9, 10]. Burguera et al. [11] give a framework to detect malware on android platform. They monitor system call in Linux level and generate software behavioral patterns and classify these patterns by using cluster algorithm. Their method is efficient in detecting malware behavior seen from Linux kernel. Unfortunately there are several malware behaviors that cannot be seen from Linux level such as malicious SMS malware or malicious call malware. They are able to track a suspicious third-party application because they used dynamic analysis techniques to monitor sensitive information on android. The drawback of the system is that many normal applications may be considered as malware. Enck et al. presented TaintDroid in [12]. Their system used dynamic analysis techniques to monitor sensitive information on android. Thus, they can track a suspicious third-party application that uses sensitive data like GPS location information or address book information. The shortcoming of their method is that an application with sensitive data may be considered as malware [8, 9, 12]. Lee et al. [13] have found that white list Smartphone environment contributes the idea of a white list server to store the identity of any application in the database so the server may recognize friendly application not as a malware. Infections are blocked by using reputation-based collected data and information. Marforio et al. [14] have been working on a coordinated attack against modern Smartphone system and this can lead to disclosure of user private data to third parties. Discussion about countermeasures can be used for protection against these kinds of attacks.

Metamorphic malware has become a subject in an ongoing and underdevelopment research from 2009. You and Yim have been contributing in the construction of malware obfuscation techniques. They explain a few general techniques in obfuscation malware [17].

## 3. Research Framework

The primary goal of this research study is to investigate the security risks associated with the use of android. It will contribute to regulate data in mobile computing on Smartphone, especially android mobile system. The proposed solution will detect attacks (viruses, worms, Trojan horses, and metamorphic malware) and prompt users to take actions to prevent breaches. Any suspicious activity that may reveal personal information to third parties or unknown entities will be reported to users to prevent potential attacks. This research study is different in that it will leverage previously proposed and implemented defense strategies and present an enhanced protection framework that will address android's vulnerabilities and risks. Furthermore, this project will extend the existing knowledge about android Smartphone's security and provide in-depth understanding of how to effectively manage emerging threats and fend off attacks, an issue that has long been realized and pointed out by security researchers and required more extensive research.

Android's threats are further amplified by the fact that users are limited to use their Smartphone for basic services and functions, such as email and SMS/MMS. Android's open-source nature further increases security vulnerabilities because cybercriminals could easily exploit this feature to modify the core applications and inserted malicious software to cause damage and monetary loss.

*Research Question*

- What are the parameters to monitor, detect, and track? From the result and related work which has already been done and still in progress, the possible parameters are to monitor all applications, through detection using static analysis code and signature behavior stored within the database that is controlled by cloud computational. By this scenario, using Smartphone to execute MDTN will become efficient though the drawback is to bypass malware because of the unavailability of behavior and new signature outside of database.
- Has the MDTN process been approved to protect sensitive data from the Smartphone user?A few ideas and implementation to detect malware have been executed, though the idea for detection using static behavior analysis that connected to cloud computational is still new. Based on research and text book about malware detection, MDTN process is logically possible and the mechanism has been executed well.
- How many malwares that could be anticipated using MDTN? For the time being, only generally known malwares are recognized by behavior signature and static analysis code.
- Has MDTN fulfilled the security requirement about confidentiality, integrity, availability, authenticity, and accountability? Confidentiality, integrity, availability, authenticity, and accountability are general requirements for security issue; thus MDTN has to fulfill these prerequisites.

## 4. Malware Behavior in Smartphone Environment

There are two methods of an intruder to steal data from Smartphone as follows.

- Trojanized apps: cybercriminals will download an app from mobile store and then reupload the app into the app site with injected malicious malware.
- Malicious apps: cybercriminals will create malicious apps under the disguise of popular mobile app and upload them to the mobile store [18].

Vennon, a GTC engineer at Smobile Systems, has stated that malware is categorized based on what the malware does once it has infected a system. The categories are as follows [19].

- Virus: a virus is defined as a destructive or malicious program that lacks the capacity to self-reproduce.
- Worm: this is a malicious code that can control system vulnerability or a network in order to automatically duplicate to another system.
- Trojan: a Trojan allows an attacker to obtain unauthorized access or remote access to a system while it appears to be executing a required operation.
- Spyware and adware: this destructive application conceals itself from the user while it collects information about the user without the user's permission.
- Phishing apps: this malware is disguising itself as a legitimate site but containing mobile phishing that could steal user credential data. Malicious application is discovered by the user after installation and infection.
- Bot processes: hidden processes can execute completely invisible to the user, run executables, or contact botmasters for new instructions. Botnet strives to hijack and control infected devices.
- Mobile malware symptoms: signs of a malware infection can include unwanted behaviors and degradation of device performance. Performance issues such as frozen apps, failure to reboot, and difficulty connecting to the network are also common. Mobile malware can eat up battery or processing power, hijack the browser, send unauthorized SMS messages, and freeze or brick the device entirely.

Schmidt et al. [20] have announced the evolution of malware up to 2008. The malicious Linux binary itself is packed as “raw resource” into this Java application, for example, as png file, which can be seen on Figure 3. After installation, the Java application has to be executed once in order to rename the resource file into the appropriate binary. After renaming the file, the file has to be made executable which is currently impossible from within Java.

Malwares have various variants; one of them is metamorphic malware. The malware uses semantics-preserving transformations (obfuscations) to change its own code as it progresses. It progresses by repeating the computing process and applying the result of previous stage so the next stage will be different from the last. Any signature-based antivirus program will find it difficult to detect the malicious malware. Despite the ongoing changes, the function stays the same. The longer the malware stays, the more it evolves, making it difficult for the antivirus to defend the system. Obfuscation is to make the information less clear and more difficult to understand. Software vendors use obfuscation technique to prevent the software from reversing the engineer. Intruders use obfuscation transformations so the malware may never reverse the engineer and the malicious intent cannot be comprehended.

*Obfuscated Code for Dead Code Insertion and Code Reordering. *Consider

(1) $$\begin{array}{c} \text{mov}\;\text{eax},\left[\text{edx}+0\text{Ch}\right],\\ \text{jmp}+3,\\ \text{push}\;\text{ebx},\\ \text{dec}\;\text{eax},\\ \text{jmp}+4,\\ \text{inc}\;\text{eax},\\ \text{jmp}-3,\\ \text{call}\;\text{Release}\;\text{Lock},\\ \text{jmp}+2,\\ \text{push}\;\left[\text{eax}\right],\\ \text{jmp}-2. \end{array}$$

*“Dead code”* is semantically equivalent to a nil operation. Insertion of this type of code has no semantic impact on the malware. The insertion increases the size of the malware and modifies the byte and instruction level content of the malware.* “Code reordering”* changes the syntactic order of the code in the malware. The actual or semantic execution path of the program does not change but only the syntactic order as present in the malware image. Code reordering includes the techniques of branch obfuscation, branch inversion, and branch flipping and the use of opaque predicates.

## 5. Proposed Idea and Design

The idea of this paper is to construct a proper android environment.  Figure 6 illustrates the flowchart of the MDTN system contains Monitoring, detecting, tracking, and notification (MDTN) which is interconnected in this proposed idea.

### 5.1. Monitoring

Scanning all application and activity in Smartphone: the engine must examine and monitor various locations of the computer such as the hard disk, registry, and main memory. If a change to a critical component is detected, it could be a sign of infection.

Third-party applications are entrusted with several types of privacy sensitive information. The monitoring system must distinguish multiple information types, which requires additional computation and storage.

System activities include any action of interest which may be taken by the system, typically utilizing system resources. When integrated with system resource monitoring, these features can be used to study how activities impact system resource usage. When integrated with user activity monitoring, these features can be used to study how user activity impacts the system.

Monitoring system can also be used to continuously monitor features but only issue callbacks when certain conditions are met. These monitors will be referred to as notifies. The monitoring module will continuously monitor these features at the requested frequency but will only initiate a call to the callback function when the specified criteria are met. The format for such a request is similar to the monitor request, but with the additional information to specify the notification conditions. Monitoring System includes application and screen activities which listed in Table 3.

Context-based privacy sensitive information is dynamic and can be difficult to identify even when sent in the clear. For example, geographic locations are pairs of floating point numbers that frequently change and are hard to predict [12].

### 5.2. Detecting

A malware detector is a system responsible to determine whether a program has malicious behavior. In other words malware detector D is defined as a function: D : A{Malware, Normal} where D is set for detecting and A is set for application. Consider

(2) $$\begin{array}{c} \text{D}\left(\text{A}\right)\{\begin{array}{cc} \text{Malware} & \\ \text{Normal}. & \end{array} \end{array}$$

Detecting process will determine whether an application is a malware or legit through recorded behavior that is in line with library detector.

Generally, there are two techinques to detect malwares: anomaly-based technique and signature-based technique. Signature-based detection techniques define every known malwares by signature or particular patterns to identify malicious program. Anomaly-based detection techniques model normal behavior during a training phase and use this normal model to identify malicious programs.

**Figure 5:**
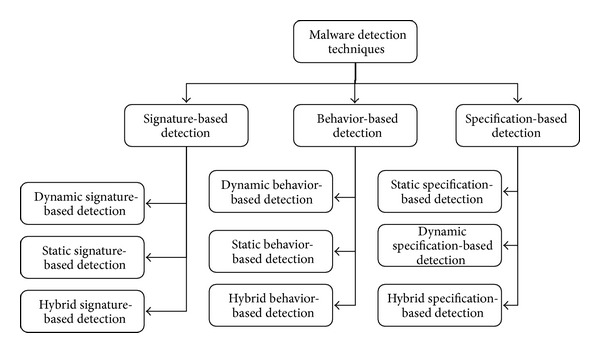
A classification of Smartphone malware detection techniques.

**Figure 6:**
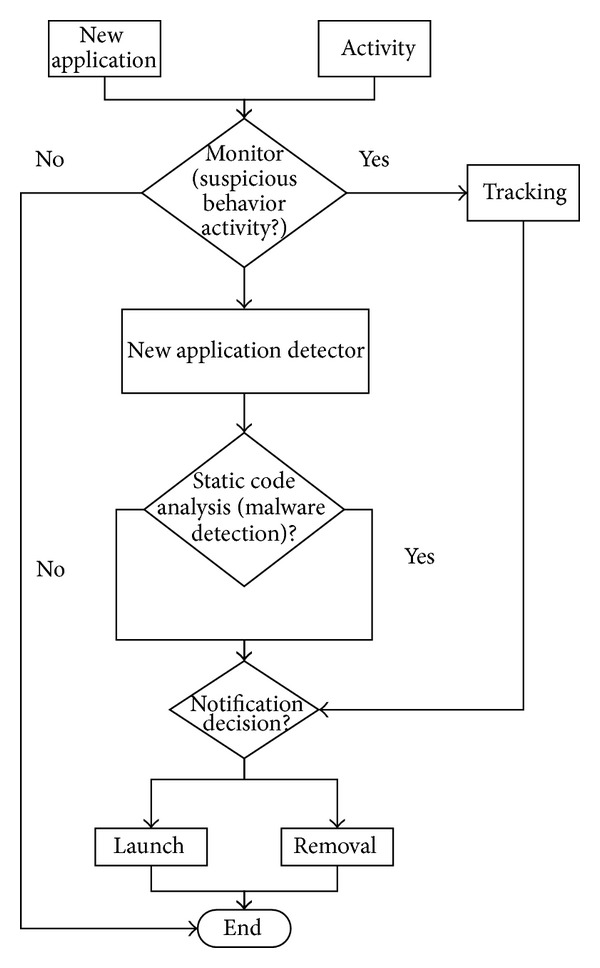
Flowchart of the system.

Figure 5 illustrates the classification of malware detection techniques. In this classification, we followed the defined three rules. Reference behavior rule classified detection techniques broadly into two main categories: anomaly-based technique and signature-based technique. An anomaly-based detection technique constructs normal behavior model during the training phase. In detection phase any deviation from this model can be considered maliciousness [15].

This detection system is using behavior-based detection. This technique is a complex metastructure with dynamic concept and semantic interpretation. Behavior-based detection is effective and efficient to deal with complex techniques, such as polymorphic, binary packers, and encryption. This method is based on static code analysis which uses information embedded in a given executable file or code templates to capture the functionality of a specific malware. Behavior-based detection techniques assume that an intrusion can be detected by observing a deviation from normal or expected behavior of the system or the users. The model of normal or valid behavior is extracted from reference information collected by various means. The intrusion detection system later compares this model with the current activity. Advantages of behavior-based approaches are that they can detect attempts to exploit new and unforeseen vulnerabilities, the advantages and disadvantages of this method listed in Table 4.

Once the engine has detected an item that requires further examination, the engine will refer to an updated list of known malware, called the “blacklist”. The blacklist contains “signatures” or identifiable patterns of know malware. The engine will be able to determine whether any filematches any of the known malware. If a match is identified, the file is classified according to the particular category: Malware's integrity identification, eradication of the particular packages, publication of the list of packages to the remote server, and so forth [21]. One of the methods to perform detection is

(3) $$\begin{array}{c} PackageInfo\;class:signatures\left(\right),\\ getInstalledPackages\left(\right). \end{array}$$

*Removal*. The final step for this engine is to take appropriate actions on files that are identified as malware. In most circumstances, the engine removes the program or file completely and restores the computer to its ore-infection state. Otherwise, a file can be disabled or quarantined, so that the user could enable it later.

*For Metamorphic Malware There Is an Interpretation for Obfuscating Solution.* Abstract interpretation declared in 1977 is a general model for the (static or dynamic) approximation of semantics of discrete dynamic systems. Obfuscating programs is making abstract interpreters incomplete. Modifying the simple self-interpreter so that all values in the store are obfuscated.  Algorithm 1 illustrates Formal framework for malware detection itself are based on program semantics and abstract interpretation. This is as follows for obfuscation interpretation in metamorphic malware.

The following are the obfuscation techniques that are particularly used by metamorphic viruses [22]:

- data flow obfuscation (instruction substitution, instruction permutation,* dead code* or* garbage code* insertion, variable substitution);
- control flow obfuscation (changing the control flow).

Modification of interpretation for obfuscation code is as follows.

- Input values are obfuscated in the initial store.
- Variable values are obfuscated just before putting in the store.
- Output values are deobfuscated in the program's final store.
- Expression evaluation yields nonobfuscated values:
  - constant values are not obfuscated;
  - variables' values must be deobfuscated when got from the store.

 Let *ρ* ∈ *uco*(Σ)* with *Σ* semantic objects (data, traces, etc.)*
 
*A program transformation τ* : *P* → *P such that *⟦*P*⟧ = ⟦*τ*(*P*)⟧
 
* *
*ρ* **β*-complete for *⟦·⟧* if ρ*(⟦*P*⟧) = ⟦*P*⟧^*ρ*^
   
* τ* *obfuscates P* *if *⟦*P*⟧^*ρ*^⊏⟦*τ*(*P*)⟧^*ρ*^
   
* *⟦*P*⟧^*ρ*^⊏⟦*τ*(*P*)⟧^*ρ*^⇔*ρ*(⟦*τ*(*P*)⟧)⊏⟦*τ*(*P*)⟧^*ρ*^
    (4) D(P,M)={true,if   D   determines  that  Pis  infected  with  M,false,otherwise.

 
*Consider a set o* *of obfuscating transformations ranged over by o*.
 Let *M*↪*P denote that program P is infected with malware M*.
 
* D* *is *sound* for  o  if  D*(*P*, *M*) = * true *⇒∃*o* ∈ *o* : *o*(*M*)↪*P*.
 
* D* *is *complete* for  o  if *∀*o*(*M*)↪*P*⇒*D*(*P*, *M*) = * true,*

where an ideal malware detector is sound and complete: sound means no false positives and complete means no false negatives.

*Certifying Malware Detecting.* We can characterize the most concrete property *ϕ* such that

 
*MDρ*(*M*, *P*) = * true *⇔∃ *Τ* ∈ *Progr* : ⟦*I*(*M*,*T*)⟧^*ρ*^ = ⟦*P*⟧^*ρ*^ *is *sound and complete for*o*_*ϕ*_.

*Training Malware Detecting.* Given *o*_*ϕ*_ we can characterize the most concrete property *ρ* such that MD is complete for *o*_*ϕ*_.

### 5.3. Tracking

Tracking is a phase where the application will track the source of the problem and perform the tracking activity in the Smartphone.

The following could be logged to represent a user:

- Secure.ANDROID_ID (has limitations),
- TelephonyManager:
  - getSimSerialNumber(),
  - getDeviceID(),
- Build.Serial (good for tablets),
- Company-assigned ID.

The process for data tracking is started from detected tainted source or suspicious behavior. Tainted data comes from specific source; thus the contaminated data shall be tracked down specifically and dealt with. After the purging process is done, the result will be reported that the decontamination has been finalized.

To track the URL location of the intruder who remotely controls the malware inside the Smartphone, the following method will be used:

(5) $$\begin{array}{c} Get\_Document\left(\right)\\ Get\_LocationURL\left(\right)\\ Get\text{-}LocationName\left(\right). \end{array}$$

The device's IMEI was also exposed by applications. The IMEI uniquely identifies a specific mobile phone and is used to prevent a stolen handset from accessing the cellular network. TaintDroid flags indicated that nine applications transmitted the IMEI.

Seven out of the nine applications either do not represent an end user license agreement (EULA) or do not specify IMEI collection in the EULA [12]. From this result, tracking IMEI and IMSI activity should be made known to users to let them determine which activity is remotely controlled by intruders.

The method for IMEI and IMSI (personal information) is as follows:

(6) Methods  of  the  TelephonyManager  class:get  DeviceId(),getSubscriberId(),getNetworkOperator(),getLine1Number(),getSimOperator(),getSimSerialNumber(),getSimCountryIso().

### 5.4. Notification

The action is defined by a PendingIntent containing an* Intent* that starts an activity in your application. To associate the PendingIntent with a gesture, call the appropriate method of* NotificationCompat.Builder.*

A PendingIntent object helps to perform an action on the application's behalf, often at a later time, without caring about whether or not the application is running. After the action is performed,* NotificationManager.notify()* is called to pass the notification object to the system by sending the particular task.

The method for getting notification is as follows:

(7) NotificationCompact.Builder.build()NotificationManager.notify()Android:name=“android.support.PARENT_ACTIVITY".

## 6. The Design System of MDTN and Discussion

**Figure 7:**
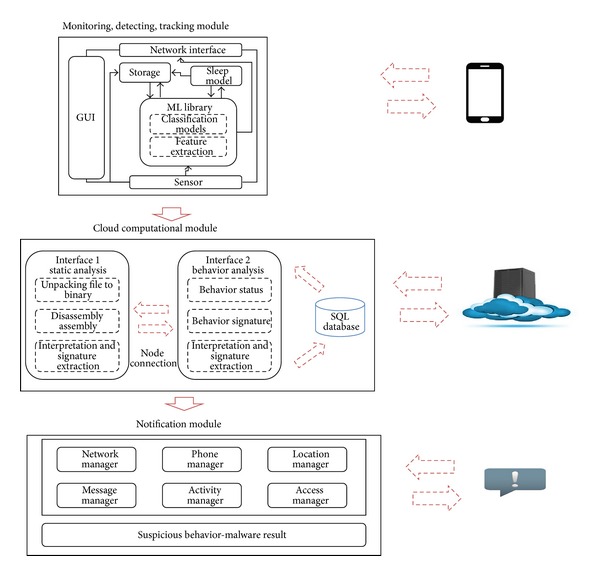
The MDTN system infrastucture.

The MDTN system is an interconnected process to monitor the downloading and installation progress of any file in a Smartphone. If in the case of suspicious behavior detected, the tracking module will be deployed to track the source of the behavior, the result will be forwarded to the notification module for the next decision. A new application which is going to be installed or web application with malware will be monitored and detected using static code analysis and signature database will determine whether the file contains any malware or not. The overall progress will be notified to the user, and the user may decide whether or not to install or to delete the given application. Figure 7 illustrates 3 modules inside MDTN system infrastructure.

There are 3 modules inside MDTN system. The first and the third module (module notification) are connected to the user, while the monitoring, detecting and tracking parts which consisted of classification models and extraction are featured inside the machine learning. They combine an internally developed platform-independent machine learning C library with specific components device—written in Java—which are responsible for communication, storage and user interface. The process is the classification pipeline which is responsible for the inference of end user behavior. The pipeline continuously samples the phone sensors and extracts features used by classification models, which also run on the phone. The classification pipeline samples one sensor, GPS. All these processes are connected with the user's smartphone.

The second module is in-between or middleware module that could reduce Smartphone's performance. Though, it has its own disadvantage when it comes to delivery process into the cloud server where the server computes the data and consumes more time to redeliver back to the user. All data is stored within independent SQLite files. These files are transferred to the cloud infrastructure with an uploading policy that emphasizes energy efficiency to minimize the impact of using the phone's batteries.

**Figure 8:**
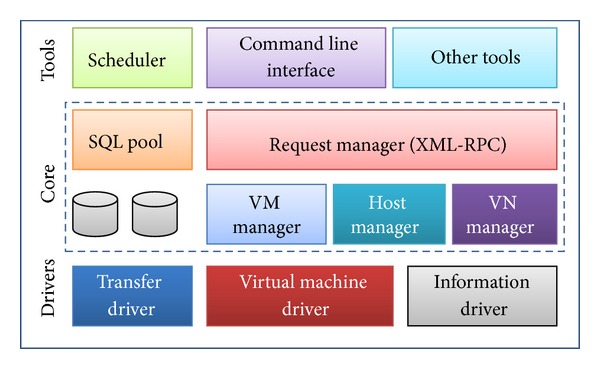
The three layers of the private cloud internal architecture.

For this case we build our own Private Cloud Eucalyptus which was bundled with Ubuntu (UEC-Ubuntu is bundling OpenStack from 11.10). UEC/Eucalyptus is an on-premise private cloud OSS based platform, sponsored by Eucalyptus Systems, Linux based–RHEL, CentOS, Ubuntu, Support for VMware. Figure 8 illustrates the 3 layers inside the private cloud internal architecture that used in this work.

## 7. Performance Analysis

A standardized measurement should first be set to test and evaluate the performance in the two systems in which total time execution is used. Basically, throughput is a decent indicator of malware analyzer performance. Within a period of time, throughput will calculate the total number of completed analysis task. Thus, the total consumed time is used in which the defined samples affect the total number submitted in a fixed number. The time is calculated by summing the in-between period of the last analysis sample and the previous analysis time within the same group sampling.

Total execution time *T*(*n*) for task *D*(*P*, *M*) consists of three elements: setup time (*t*_*s*_), execution time (*t*_*e*_), and postprocessing time (*t*_*p*_). Setup time is the total time to prepare and to deploy the required accessible malware sample. The time in the second system will become the connector as it requires longer time comparing to the first system. Nevertheless, the execution and postprocessing time itself will be the same in the first and the second systems. The setup time itself is a drawback to the second system, thus giving advantage to the first system which practically uses cloud computing. The required records from each tasks needed to calculate *T*(*n*) are submit time, start analysis time, and finish time.

### 7.1. Mobile Malware List (Table 5)

This implementation is going to be executed on android OS platform using Samsung Galaxy S3, android version 4.3, IMEI number 352905053490342. This research uses 8 of biggest attack percentage mobile software, and this research uses 12 different types of mobile software.

### 7.2. System Performance Evaluation (Table 6)

12 types of mobile software (.APK) under trial are successfully detected as malware by the system on this system performance. Stabilized time (ms) is the time needed for the system to recognize the mobile software as a malware and the notification process via executable binary file. Total stabilized time *T*(*n*) consists of three elements: setup time (*t*_*s*_), execution time (*t*_*e*_), and postprocessing time (*t*_*p*_).

### 7.3. Tracking System Performance (Table 7)

The tracking phase is not maximally carried out, as the result services of Zitmo android do not show and GoldDream's server is not detected. Under services, any service that is going to be used by the intruders can be tracked (data stealing). From the tracking result, user is able to know which of the many services on android system is under monitoring or modification, or under threat from the particular server. The tracking has not been maximally carried out by scrutinizing any server that serves the intruders, and so far tracking depends on intruder's known website. In the future, the malware's server can be reported to the database system so that the particular application can be blocked.

### 7.4. Comparison System Performance (Table 8)

On comparison with the 3 previous projects that have been carried out and developed—like TaintDroid, CrowDroid, and RoboDroid—real time monitoring and tracking system is provided by TaintDroid by which the kernel level is exercised. TaintDroid is an android operating system with added real time monitoring and tracking system.

## 8. Conclusion and Future Work

MDTN is an interconnected system process for Smartphone environment. For this research paper, the author uses Android OS because the operating system is frequently attacked by cybercriminals. Monitoring, detecting, tracking, and notification are used not only to check new application before being installed into the Smartphone, but also to detect suspicious behavior activity in real time. As for the detection method, behavior-based detection technique and database static code analysis are used to determine suspicious behavior and malware application. The tracking part can be developed to later stage for the purpose of preventing future threat realization. In the case of reoccurrence, the system is able to block and recognizing the data as spam or threat.

## Figures and Tables

**Algorithm 1:**
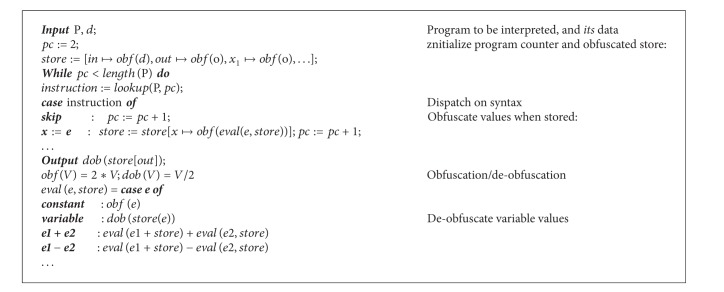


**Table 1:** Market share among Smartphone OS-IDC worldwide quarterly mobile phone tracker, May 2013.

Operating system	2012 market share (%)	2013 market share (%)	Year over year change (%)
Android	59.1	75.0	79.5
iOS	23.0	17.3	6.6
Windows phone	2.0	3.2	133.3
Blackberry OS	6.4	2.9	−35.1
Linux	2.4	1.0	−41.7
Symbian	6.8	0.6	−88.5
Others	0.4	0.0	−83.3

**Table 2:** Modification of threats among Smartphone OS-IDC worldwide quarterly mobile phone tracker, May 2013.

Platform	Modification	Family
Android	43600	255
J2ME	2257	64
Symbian	445	113
Windows mobile	85	27
Others	28	10

Total	46415	469

**Table 3:** Monitoring features for user activity.

Activity	Feature	Description
Application	appopen appclose	Open/close of application by user
Screen	screen	On/off screen

**Table 4:** Advantages and disadvantages of behavior signature-based technique [15].

Advantages	Disadvantages
Fast and safe	Difficulty analyzing unknown malware
Low level of false positives	Cannot detect unknown malwares
Good in analyzing multipath malware	Not able to detect a lot of polymorphic viruses present (Packers)
Detect entire family of malware with one signature	
Detect malware before its execution	
Best results in detecting of polymorphic malware	

**Table 5:** Mobile malware for this research.

Name	Package's name	% of attacks
Trojan-SMS	Android SMS Trojan, jSMSHider	33.5
Backdoor	Obad	20.6
Trojan	Crazyapps angry.birds, Beauty.Girl-1	19.4
Adware	Airpush-Minimob	7.1
RiskTool	Mobile Spy	6.0
Trojan-Downloader	Trojan_Extension	5.8
Trojan-Spy	Andr/PJApps	4.0
Others apps	DroidKungFu, Zitmo android, GoldDream	3.6

**Table 6:** System performance result.

Virus source	System performance		
	Detection	Memory remains (%)	Time (s)
Android SMS Trojan	Yes	85.69	5.24
jSMSHider	Yes	92.63	3.50
Obad	Yes	79.61	6.26
crazyapps.angry.birds	Yes	87.38	4.25
com.Beauty.Girl-1	Yes	89.45	4.26
Airpush-Minimob	Yes	78.26	6.02
Mobile Spy	Yes	86.24	5.63
Trojan_Extension	Yes	78.86	6.24
Andr/PJApps	Yes	95.25	5.23
Zitmo android	Yes	84.12	4.35
DroidKungFu	Yes	93.14	4.58
GoldDream	Yes	80.21	4.53

**Table 7:** Tracking result.

Name	Tracked server	Services
Android SMS Trojan	*✓*	Phone Number, IMEI, IMSI
jSMSHider	*✓*	SMS, MMS transaction
Obad	*✓*	MMS transaction, admin system
crazyapps.angry.birds	*✓*	Music MediaPlayback, MMS transaction
com.Beauty.Girl-1	*✓*	Music MediaPlayback, MMS transaction
Airpush-Minimob	*✓*	IMEI, MMS transaction
Mobile Spy	*✓*	MMS transaction, MediaPlayback
Trojan_Extension	*✓*	IMEI, IMSI, MMS transaction, MediaPlayback, CoreService, EmailService, CheckingService, bluetooth, SMSReciever
Andr/PJApps	*✓*	Music MediaPlayback, system security
Zitmo android	*✓*	—
DroidKungFu	*✓*	Work service, MMS, email
GoldDream	—	MMS transaction

**Table 8:** Comparison performance.

Characteristic	TaintDroid [12]	CrowDroid [11]	RobotDroid [16]	MDTN
Detection techniques	Anomaly detection	Behavior based-dynamic analysis	Support vector machine active learning algorithm	Behavior based-static analysis
Operating system	TaintDroid	Android	Android	Android
Kernel level	Yes	No	No	No
Real time monitoring	Yes	No	No	Yes
Tracking system	Yes	No	No	Yes
